# Data Ecosystems for Scientific Experiments: Managing Combustion Experiments and Simulation Analyses in Chemical Engineering

**DOI:** 10.3389/fdata.2021.663410

**Published:** 2021-09-15

**Authors:** Edoardo Ramalli, Gabriele Scalia, Barbara Pernici, Alessandro Stagni, Alberto Cuoci, Tiziano Faravelli

**Affiliations:** ^1^ Department of Electronics, Information and Bioengineering, Politecnico di Milano, Milano, Italy; ^2^ Department of Chemistry, Materials, and Chemical Engineering Giulio Natta, Politecnico di Milano, Milano, Italy

**Keywords:** scientific experiments, experiments management, data management, data validation, simulation analysis, scientific model development, data quality, combustion kinetics

## Abstract

The development of scientific predictive models has been of great interest over the decades. A scientific model is capable of forecasting domain outcomes without the necessity of performing expensive experiments. In particular, in combustion kinetics, the model can help improving the combustion facilities and the fuel efficiency reducing the pollutants. At the same time, the amount of available scientific data has increased and helped speeding up the continuous cycle of model improvement and validation. This has also opened new opportunities for leveraging a large amount of data to support knowledge extraction. However, experiments are affected by several data quality problems since they are a collection of information over several decades of research, each characterized by different representation formats and reasons of uncertainty. In this context, it is necessary to develop an automatic data ecosystem capable of integrating heterogeneous information sources while maintaining a quality repository. We present an innovative approach to data quality management from the chemical engineering domain, based on an available prototype of a scientific framework, SciExpeM, which has been significantly extended. We identified a new methodology from the model development research process that systematically extracts knowledge from the experimental data and the predictive model. In the paper, we show how our general framework could support the model development process, and save precious research time also in other experimental domains with similar characteristics, i.e., managing numerical data from experiments.

## 1 Introduction

One of the characteristics of Industry 4.0 is the availability of vast amounts of experimental data that facilitates the development and refinement of predictive models. Such models are fundamental to speed up the development process, as they allow analyzing the characteristics of systems being designed and the properties of objects, thus improving their quality. Consequently, the need emerged to systematically store and manage large quantities of experimental data collected and shared by various stakeholders. Such Data Ecosystems (cfr. Cappiello et al. (2020)) present several challenges and advanced requirements, ranging from the need to reconcile different and sometimes incompatible representations of data, assessing and guaranteeing agreed quality levels, and preserving property rights while sharing data.

Such systems are being discussed in the Industry 4.0 domain (cfr. Jarke et al. (2019)), as a pillar for improving different industry sectors, e.g., in the automotive, airline, or machine-building industries. In particular, in supply chains, lengthy negotiations for agreeing on formats of shared data and their management should be semi-automatically negotiated, executed, and monitored for contractual and legal compliance. As a result, frameworks for data exchange are supported by data analysis capabilities and data quality assessment tools. In general, data re-purposing for analysis and the development of models with AI technologies require a mutual understanding of the data and their associated characteristics.

In this context, there is a need to define an automated and standardized procedure that limits the errors in the collected experiments but at the same time responds to the characteristics of Data Science applications on big data such as large volume, acquisition speed, and variety, as described by George et al. (2016), while maintaining the FAIR (Findable, Accessible, Interoperable, Reusable) policy requirements (cfr. Wilkinson et al. (2016)).

In addition, it is clearly emerging how data management, while necessary, it is only a precondition in Data Science. For instance, Stodden (2020) advocates the need of supporting the Data Science life cycle with adequate tools and considering it a process composed of different phases for which different strategies and tools are needed. First of all, it is necessary to clearly define the roles of users in such processes and the sharing modalities. As discussed in Curry and Sheth (2018), in new data-driven smart systems the participants can achieve results that would not be achievable by each participant in isolation. As discussed in the paper, the pillars of such systems are, in addition to data management, adaptivity and user interactions support, and all are based on trusted data platforms to enable data sharing. Different models of interdependence among participants can emerge, depending on the level of control of the participants on data and the relationships among participants. Current approaches follow a so-called “Directed data ecosystem”, according to the typologies defined in Curry and Sheth (2018), as they are centrally controlled to fulfill a specific purpose. The envisioned evolution is toward “Virtual data ecosystems” to pool resources towards specific goals.

In the present paper, we discuss these concepts in the context of the development of prediction models and simulators in fuel analysis, in the numerical combustion domain. This sector aims to minimize the environmental impact of combustion in terms of greenhouse gas emissions and pollutants formation, improving the combustion efficiency and defining the optimal operating conditions (temperature, pressure, composition) for each fuel. Even the most negligible improvements in fuel consumption would result in huge savings of fuel itself and parallel reductions in gas and particles polluting emissions. Developing reliable kinetic models describing the combustion behavior in the broadest range of operating conditions is necessary to achieve these goals. These models are essential at the industrial level to design industrial reactors and burners, minimizing pollutant emissions.

Existing tools focus on analyzing small sets of experiments and comparing them with the results of models, also managing uncertainty in the experimental data. In contrast, it has been shown how the large-scale analysis of multiple experiments/models can drive the discovery of new knowledge (see, for example, Hansen et al. (2018)). Therefore, automatic tools and repositories for systematic, large-scale analysis of multiple experiments and models hold great promises for automatic discovery and knowledge extraction.

In fact, being able to automatically simulate and analyze a large number of experiments and models, overcome the limitation of manual analysis, and detect systematic features or errors in the model or the data, as keep track of the evolution of the model itself through different versions. As a side result, the overall procedure to develop a predictive model is standardized and reduced in the time needed since the most time-consuming phases are automated.

Several challenges are arising from the construction of such a system. A major limitation is the quality of the experimental data, subject both to actual experimental errors and errors due to misrepresentation, data entry errors, and lack of data about the experiment itself (i.e., metadata), such as uncertainty values (cfr. Pernici et al. (2021)). Scientific data repositories are information systems, which are subject to well-known and analyzed data quality problems (cfr. Batini and Scannapieco (2016)). In addition, the amount of available data in the last few years has significantly increased due to more sophisticated technical equipment available for performing the experiments, also introducing more heterogeneity.

This paper presents the SciExpeM (Scientific Experiments and Models) framework as a collection of services to automatically collect, manage, and analyze experimental data and models, starting from a first prototype (cfr. Scalia et al. (2019)). In particular, we focus on new aspects, detailed in the following. The first aspect regards the design of the new services necessary to represent more completely the research process. We advocate the importance of supporting the analysis process in all its phases. In this direction, we integrate services to improve the data quality and categorize and dynamically interpret experiments to automate the downstream analyses. To this purpose, we propose a framework that supports both storing experiments and providing services that can be combined to create flexible analysis processes keeping the possibility of defining user roles based on the necessity. To increase the framework’s flexibility, a microservice-based architecture is proposed, together with a data analysis library, to facilitate the rapid development and modification of analysis tools and services for uploading, verifying, and visualizing the data, without the need of building new GUIs (Graphical User Interfaces) to extend the system with new functionalities. Therefore, an agile development process is advocated, constructing and managing a SciExpeM service library for this purpose, demonstrating the effectiveness through several use cases.

The paper is structured as follows. In Section 2, we discuss related work and open problems. In Section 3, we present the main goals of data analysis in the numerical combustion sector, we discuss a methodological approach for the analysis process, and we introduce our framework for representing experiments, models and experiment analysis services. In Section 4 we illustrate the main components of the developed SciExpeM framework, focusing on services to support the data analysis life cycle. In Section 5, we illustrate some first results of the application of the framework, discussing in Section 6 the advantages of the approach and its current limitations and possible research directions. Finally, we summarize our work in the conclusions.

## 2 Related Work

As profoundly discussed by the Tenopir et al. (2015) survey, data sharing is an essential factor of modern scholarly debate. In recent years there has been a steady growth in the tendency to share scientific data, which can vary by discipline, age, and geographical location of the research group. The main thrust in data sharing is the benefit it derives from citations of other works, as shown in the Piwowar and Vision (2013) study, where generally, a work that shares data receives about 10*%* more citations than another equivalent work. Faniel et al. (2015) discussed that the sharing of scientific datasets allows the reuse of resources and increases the dataset’s quality since it is used and verified in various ways by different entities. However, as data sharing grows, the risks associated with it grow accordingly. It is often difficult to agree on the structure and management of the data and the infrastructure required for its sharing.

Several initiatives are being proposed for enabling data sharing and reuse in the scientific communities. Initiatives such as EOSC (European Open Science Cloud)^1^ and the recent NIST proposal for a Research Data Framework RDaF^2^ are going in the direction of providing a well-defined infrastructure for sharing scientific data and tools for their analysis. In particular, RDaF emphasizes the importance of considering the data management process in all its phases and defining its stakeholders and goals.

Clowder (cfr. Marini et al. (2018)) and Homer (cfr.Allan et al. (2012)) are an example of a framework inside EOSC to visualize and manage data but lacks a systematic methodology for handling scientific experiments together with predictive models in order to extract value from them automatically. As stated in Mirzayeva et al. (2018), “Methods for determining whether or not the model predictions are consistent with experimental data have been of great interest in combustion research over decades.” PrIMe was one of the first attempts to build a systematic repository of experiments in the combustion domain (cfr. Frenklach (2007)). PrIMe includes a database, that can be used to perform selective analyses of experiments to validate reaction model parameters as discussed in Mirzayeva et al. (2018). In particular, research has focused on analyzing the data features, with emphasis on uncertainty of experimental data. Several other repositories have been proposed as a basis for comparing models from results of experiments. ChemKED^3^ (cfr. Weber and Niemeyer (2018)) provides tools to create databases of thermodynamic data and chemical reactions, managing data consistency. It provides tools to retrieve experiments and compare them with provided models. CloudFlame^4^ (cfr. Goteng et al. (2013)) provides research tools and a database for the combustion community. The database includes digitized data of fundamental combustion experiments published in journals. ReSpecTh^5^ (cfr. Varga et al. (2015)) collects experiments (REaction kinetics, SPEctroscopy and THermochemistry experiments) and tools to analyze them, including automatic model validation (such as those used in the analysis of Olm et al. (2015)).

Existing tools focus on analyzing small sets of experiments and comparing them with the results of models, also managing uncertainty in the experimental data. In contrast, it has been shown how the large-scale analysis of multiple experiments/models can drive the discovery of new knowledge (see, for example, Hansen et al. (2018)). Therefore, automatic tools and repositories for systematic, large-scale analysis of multiple experiments and models hold great promises for automatic discovery and knowledge extraction.

In previous work of some of the authors (cfr. Scalia et al. (2018; 2019)), we discussed the requirements for a framework that goes into this direction. We discussed which services are essential for an information system storing scientific experiments, integrating them, managing their quality, their interpretation, and performing different types of data analysis both on the stored experimental data and on the results of numerical combustion models. Scalia et al. (2019) discussed the primary services, the functional requirements, and the first architecture to support the needs emerging from the large-scale data-driven validation of scientific models. Moreover, it presented the first version of SciExpeM, which has been further developed in this work. In the present paper, we introduce new types of services based on the SciExpeM service-based architecture, focusing on the systematic and automatic support of the data science process and on data quality analysis. For this purpose, we have defined roles and privileges for the different types of users who interact in the system, focusing on developing a data analysis pipeline with a human-in-the-loop process. To improve the scientific repository’s quality, we propose using simulation-based prediction techniques as a basis for data cleaning procedures.

## 3 Data Analysis in the Numerical Combustion Sector: A Methodological Approach

This section introduces the numerical combustion application domain discussed in this paper with its peculiarities and challenges (Section 3.1), presenting the general approach to developing a scientific model (Section 3.2), and identifying the roles and processes involved (Section 3.3). We describe the SciExpeM framework (Section 3.4), which is actively involved in the model development process to speed up and automate some procedures.

### 3.1 Combustion Experiments

The development of more and more accurate predictive models (cfr. Kuhn and Johnson. (2013) as a result of the technology boost in the capability of performing experiments currently drives research within the combustion community, as reviewed by Mishra (2014). However, it also affects the most various research fields, e.g., biology (cfr. Queen et al. (2002)) and climatology (cfr. Brázdil et al. (2005)). Regardless of the involved field, such experiments share the same basic limitations, i.e., the presence of an intrinsic degree of uncertainty as illustrated by Moffat (1988), and, very often, the lack of structure into user-friendly databases due to the incremental addition of experiments over time.

Concerning uncertainties, only the most recent papers (approximately after 2000) systematically report uncertainties. Indeed, their rigorous evaluation (in absolute or relative terms) would require a repeated evaluation of the same measurement for a sufficiently high number of times to have statistically significant data. In the past, this was not an easy task due to the high amount of time and economic resources to perform a single evaluation, and only recently technological progress has made this more affordable. Table 1 shows an example of large uncertainty present in experimental data, although the experiment file does not explicitly report it. At almost identical temperature and pressure conditions, there is, for example, a variation of the measured Ignition Delay Time (IDT) of over 65*%* from the measurement point of (930 *K*, 3.586* atm*) and (930 *K*, 3.534* atm*). Therefore, uncertainty is always present in all experimental data, but it is often not reported, although it is significant.

**TABLE 1:** Example of uncertainty in DOI:10.24388/g00000007 experiment by Pang et al. (2009). In bold and underlined groups of nearby points but with significantly different measured Ignition Delay Time (IDT).

Temperature [K]	Pressure [atm]	Ignition delay [us]
**930**	**3.586**	**7,912**
**930**	**3.534**	**13,090**
…	…	…
938	3.651	7,723
938	3.535	6,973
938	3.52	6,133

Regarding data organization and standardization, experiments’ heterogeneity is the main limitation to creating a uniform, easy-to-use database. Although the first classification in combustion experiments can be made according to the experimental facility (Table 2), very often, each of them exhibits unique features (e.g., temperature/pressure boundary conditions, heat losses, radiation, the definition of inlet composition), such that the definition of a standardized database requires the inclusion of a significant number of rules and exceptions. Besides, an experiment’s property can be measured using different experimental facilities requiring the definition of more rules to distinguish every case as we can see from Table 2.

**TABLE 2:** Experimental facilities needed to measure a certain property as IDT, Laminar Flame Speed (LFS), and speciation.

Experimental facility	Properties
Shock Tube	IDT, Speciation
Rapid Compression Machine	IDT
Jet Stirred Reactor	Speciation
Flow reactor	Speciation
Premixed laminar flame	LFS, Speciation
Counterflow diffusion flame	Speciation

Finding common features among the different experiments is a necessary first step to categorize them. In combustion, two major aspects can be leveraged:

1. The hierarchy of the physical process, according to which the combustion mechanism of the larger molecules depends on the smaller ones.
2. The operating conditions in which experiments are performed.

The first point greatly simplifies the issue since it creates a hierarchical and modular dependence of experiments and the related models. Therefore, for example, the combustion features of *n*-heptane, i.e., one of the main components of gasoline, are strictly dependant on those of hydrogen, since larger molecules break out into smaller ones; on the other hand, the opposite is not valid, and hydrogen chemistry can be decoupled from *n*-heptane one. Of course, the same applies to the related models, and hierarchical decomposition of chemical mechanisms is a common practice to set up kinetic models, as done by Ranzi et al. (2012). Second, classifying them according to operating conditions allows a critical understanding of model performance, as well as of its weaknesses, i.e., regions of the domain where model improvements are needed, or experiments need to be further refined to be able to validate more carefully the model (i.e., a model-guided design of experiments, as reviewed in Franceschini and Macchietto (2008)).

In general, it is not possible to define quality-related parameters to exclude a priori an experiment to be inserted in the repository. Each experiment could have several problems, but since the experiments still have a scientific relevance in this domain, it is essential to be tolerant also to cases, for example, with an unknown uncertainty or incompleteness of information. For example, the completeness of an experiment could be addressed by dedicating additional resources to investigate the missing information. In the case of uncertainty, it will be the predictive model itself together with a similarity index to quantify the experimental data reliability.

#### 3.1.1 Experiments Acquisition

The first major obstacle in creating a structured database is collecting experiments from decades of research in different technological conditions. For methane, one of the first hydrocarbons investigated experimentally and theoretically, the first studies were performed before and after WWII, e.g. Norrish and Foord (1936); Burgoyne and Hirsch (1954). As one can imagine, the degree of accuracy of the experimental facilities where the oldest studies were performed is quite harsh to estimate.

All the experimental facilities mostly used for data acquisition (Table 2) are designed with the common principle of approximating ideal fluid dynamic conditions: since the target of the investigation is the mixture chemistry, in most experimental facilities (Shock Tube, Rapid Compression Machine, Jet Stirred Reactor, Flow Reactor) the geometry is simplified as much as possible in order to guarantee ideal conditions, i.e., a uniform temperature, pressure and composition such that the role of transport can be neglected. Despite the impressive acceleration in the measurement capabilities of the current reactors, the number and typology of sampled data points need to be designed upstream since the costs of reactor (i) operation, (ii) maintenance, and (iii) safety are often significant.

In summary, throughout the whole workflow, (Section 3.3), going from the experimental facility to the implementation into the database, the acquisition of datasets must account for the following independent sources of uncertainties, which can be characteristics of many experimental domains:

• *Uncertainty of the measurement* is an unavoidable feature of any experimental observation. It results from the accuracy of all the device components (e.g., flow meter, gas chromatograph, thermocouple, and so on) and should always be estimated before extensively using the facility. The same experiment should be repeated a sufficiently high amount of time, in accordance with the times and costs of the procedure. Data variance should be then evaluated (assuming a normal distribution).
• *Non-ideality of the facility* is often the source of systematic errors which, if not recognized *a priori* and removed, can be incredibly misleading for model validation.
• *Data acquisition from literature*, especially for the oldest datasets, when importing data from non-digital sources, the extraction procedure with or without human intervention may add further uncertainty to the values added into the database.

### 3.2 Model Evolution

The creation of experimental repositories supports the development and validation of predictive kinetic models describing the pyrolysis and oxidation of the different fuels. For this purpose, the hierarchical structure of combustion mentioned in Section 3.1 is particularly helpful to select the subset of the database to be used for model validation: using a modular approach in both model construction and repository development allows excluding from such validation models and experiments situated at a higher hierarchical level, as described by Pelucchi et al. (2019). Despite this simplification, the remaining amount of datasets to be compared against can still be overly large for a manual benchmark of a kinetic model. Moreover, considering the strong non-linearity of chemical kinetics, the kinetic mechanism improvement could hardly find convergence to a satisfactory level if the validation is qualitative, i.e., based on the subjective judgment of the kinetic modeler, who overlaps experimental data points and modeling predictions. Indeed, two major drivers make this validation process dynamic and iterative, rather than static: (i) the availability of newer experimental measurements, extending the benchmark pool of the kinetic model, and (ii) the substantial recent advancements in the field of quantum chemistry as explained by Klippenstein (2017), paving the way to a more accurate fundamental evaluation of the kinetic parameters constituting the kinetic model. As a result, the update process of a kinetic mechanism is a “continuous-improvement” methodology.

**FIGURE 1:**
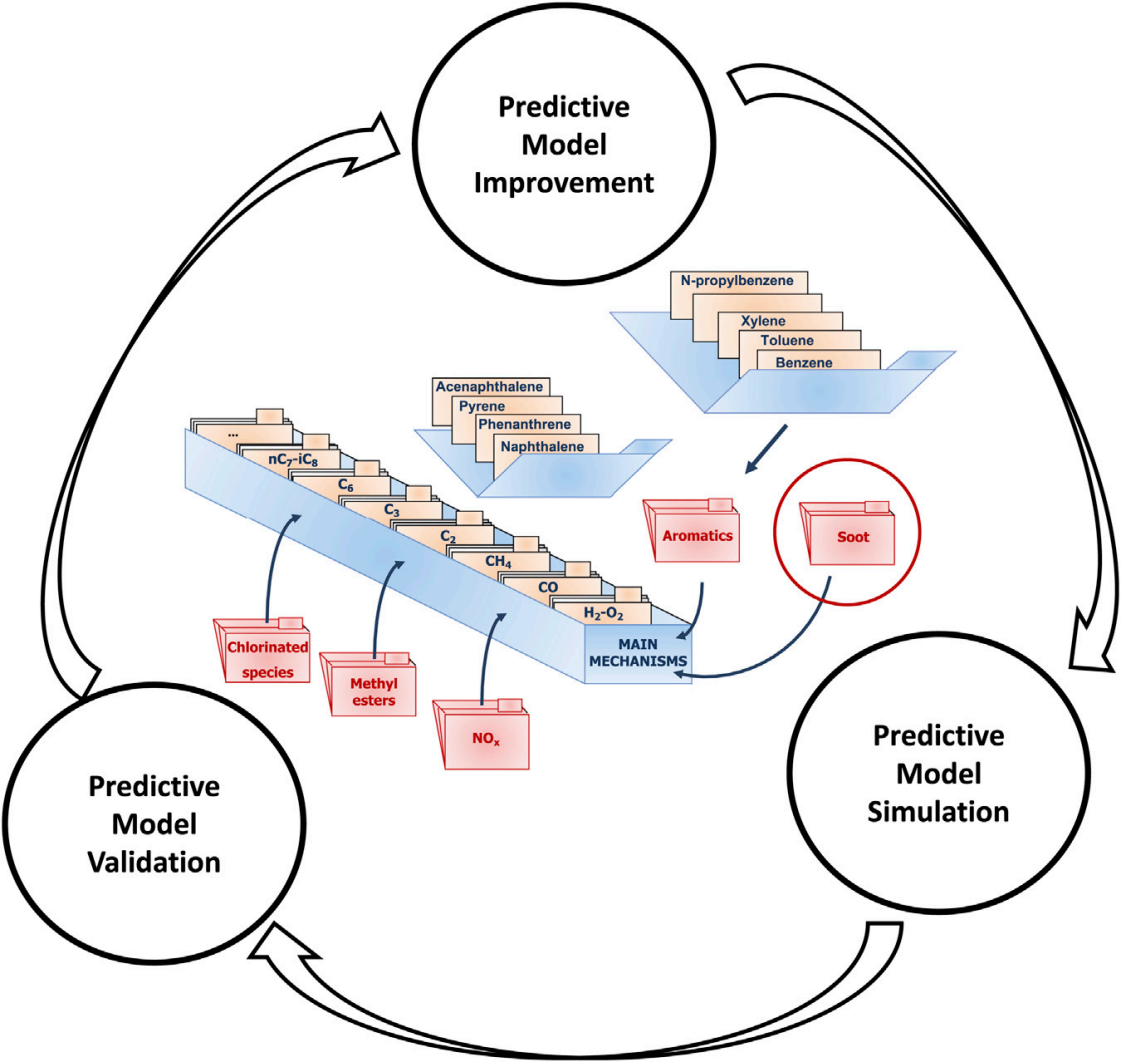
Continuous-improvement process of the CRECK predictive kinetic model - Ranzi et al. (2012).

Figure 1 illustrates the workflow, as applied to the CRECK predictive kinetic model, formulated following a hierarchical and modular methodology. This is composed of three separate steps, connected together through a continuous-improvement methodology: i) after the predictive model is developed, using the proper numerical tools, it is used to simulate the available experimental datasets. ii) Afterwards, the output obtained from the numerical simulations is compared to the experimental datasets, and a quantitative answer about the accuracy of such predictions is provided (cfr. Section 3.2.1). iii) Finally, according to the results of the validation phase, the model is updated to improve the predictions in the weak performing areas, and the loop is restarted to check again the model performance against the whole experimental database.

#### 3.2.1 Model Validation

In order to provide an objective, quantitative answer to the agreement between experiments and models, a score-based procedure must be devised, able to provide a global assessment of model performance *via* a single value. With this ultimate target, the *Curve Matching* similarity measure was first devised by Bernardi et al. (2016), and the most recent version, implemented in this work, can be found in the work by Pelucchi et al. (2019). The Curve Matching provides an index in the [0,1] range to evaluate how close the results of a simulation are to the experimental data, taking into account also the uncertainty of experimental data.

The methodology is based on the functional analysis, whose basic principles are reported in Ramsay (2004), of both the experimental data points and the model predictions, performed through a spline-based interpolation of both data series. A smoothing procedure is carried out by adopting a roughness penalty on the second derivative. Such penalty is weighted via a generalized cross-validation methodology, applied on the first derivative of the function. In this way, continuous functions, as well as their derivatives, can be compared between each other.

Traditionally, literature has always quantified the agreement between experimental data points and related predictions by assessing the sum of squared deviations between them, as done for example by Olm et al. (2014), Olm et al. (2015). This approach is quite immediate to be implemented. Yet, the obtained index value does not provide any information about the source of the deviations, since it does not consider the shape of the curves. On the other hand, including them in the model classification allows for a comprehensive, multifaceted analysis of the differences between experiments and modeling. Moreover, the value provided by the sum-of-squared deviations is only valid in relative terms, i.e., it classifies model predictions only compared to other models.

The *Curve Matching* similarity measure solves both issues by laying its foundations on three major cornerstones:

• The evaluation of distance indices, based on *L* ^2^ norm, and similarity indices, based on the Pearson Correlation, for both functions and the related derivatives. Doing so, the difference in shape can be accounted for, too, while still keeping the information about the sum-of-squared deviation *via* the distance index between experiments and models.
• The formulation of such indices to be bounded between 0 and 1, where the boundaries indicate the maximum dissimilarity and similarity, respectively. In this way the agreement of a given model can be assessed independently from the presence of other comparisons.
• The evaluation of a shift index, evaluating the differences between experiments and models in the horizontal direction, too.

In this way, a global index is returned by averaging the performance indices, thus giving an overall evaluation of the model accuracy, for which the separate analysis of the indices allows identifying the source of the deviations.

A detailed description of the approach is available in the work by Pelucchi et al. (2019). Here, the cornerstones of the approach are recalled. Four indices are conceived in such a way to be constrained between 0 and 1, with the two boundaries representing maximum dissimilarity and similarity, respectively. Such indices are based on the following definitions:

• *f* and *g*, i.e. the functional curves representing experiment and model data points, respectively, and *f*′ and *g*′ their derivatives
• *D*, i.e. the intersection of the domains of *f* and *g*
• $\left\|h\right\|$, i.e. the norm of a generic curve *h* in the *L* ^2^ space:

$$\left\|h\right\|=\sqrt{\int_{D}h{\left(x\right)}^{2}dx}$$ (1)

The dissimilarity indices are respectively introduced as:

$$d_{L_{2}}^{0}\left(\;f,g\right)=\frac{1}{1+\frac{\left\|\;f-g\right\|}{D}}\in \left(0,1\right)$$ (2)

$$d_{L_{2}}^{1}\left(\;f,g\right)=\frac{1}{1+\frac{\left\|{\;f}^{\prime }-g^{\prime }\right\|}{D}}\in \left(0,1\right)$$ (3)

$$d_{P}^{0}\left(\;f,g\right)=1-\frac{1}{2}\left\|\frac{f}{\left\|\;f\right\|}-\frac{g}{\left\|g\right\|}\right\|\in \left(0,1\right)$$ (4)

$$d_{P}^{1}\left(\;f,g\right)=1-\frac{1}{2}\left\|\frac{f^{\prime }}{\left\|{\;f}^{\prime }\right\|}-\frac{g^{\prime }}{\left\|g^{\prime }\right\|}\right\|\in \left(0,1\right)$$ (5)

Equation 2 can be considered as the functional version of the error function value based on the sum-of-squared deviations used in literature to compare models and experiments Olm et al. (2014, 2015). Yet, limiting the comparison to such value would not take into consideration the shape of the curves. Indeed, the remaining three indices exhibit the following properties:

$$d_{L_{2}}^{1}\left(\;f,f+a\right)=1\;\forall \;a\in R$$ (6)

$$d_{P}^{0}\left(\;f,f\times a\right)=1\;\forall \;a\in R$$ (7)

$$d_{P}^{1}\left(\;f,f\times a+b\right)=1\;\forall \;a,b\in R$$ (8)

These properties related to the respective indices Eqs 3–5 quantify the trend similarity of two curves in terms of a vertical shift (Eq. 6), a dilation (Eq. 7), or a dilation and a vertical shift (Eq. 8). As a fifth criterion, the horizontal shift between model and experiments is calculated as:

$$S=\max\left(1-\frac{\delta }{D},0\right)\;\in \left(0,1\right)$$ (9)

where *δ* is the shift amount of the model, chosen as the one maximizing the sum of Eqs 2–5:

$$\delta ={\text{argmax}}_{\delta }\left(d_{L_{2}}^{0}+d_{L_{2}}^{1}+d_{P}^{0}+d_{P}^{1}\right)$$ (10)

The global index *M* evaluating model performance is evaluated as the averaged sum of the values evaluated in Eqs 2–5, recalculated after the shift is completed, and (9):

$$M=\frac{d_{L_{2},shift}^{0}+d_{L_{2},shift}^{1}+d_{P,shift}^{0}+d_{P,shift}^{1}+2S}{6}$$ (11)

As one can imagine, the reliability of this evaluation depends on the uncertainty of the different measurements, and a correlation between the uncertainty of data points and uncertainty of the global index *M* (11) is necessary. For this purpose, a bootstrapping procedure, as described in Ramsay (2004), is performed to complete the analysis: by taking into account the uncertainty of the single data point, a sufficiently high number of random values is generated, assumed as normally distributed with an average value corresponding to the data point and a standard deviation equal to the related uncertainty. Usually, some tens of random data points are sufficient to reach a statistical significance.

As a result, a number of curves equal to the number of data points can be obtained, and a global index value *M* (11) can be evaluated for each of them. The related average value and deviation provide the final benchmark about the model-experiment agreement, and uncertainty range of such result. Such a range allows identifying the degree of significance of the different models’ performance against the same experimental dataset, which is then defined as relevant only when the related uncertainty range are not overlapped between each other.

### 3.3 Research Process

As discussed in the introduction, the development of a scientific model foresees the interconnection at some points of two distinct processes. The first process concerns the actual development of the predictive model. As described in Section 3.2, the process in Figure 1 is a continuous cycle of improvement, simulation, and validation of the predictive model. The second process regards the life cycle of the experiments. In reality, these procedures are more complex and interconnected, and this section aims to provide more details on the various stages.

The process that concerns the *life cycle of the experiments* includes two macro phases, which are then divided into more specific tasks as follows:

1. *Experiment Collection.* In order to validate the developed module, we need experimental data. The collection of experiments can take place in two ways:
  a. *Existing Experiment.* The researcher can use experiments existing in the literature or coming from private communications between research laboratories.
  b. *Non-Existing Experiment.* The researcher requests to perform an experiment that does not exist yet (Design of Experiment).
2. *Experiment Curation.* Once an experimental campaign on a specific fuel that describes different characteristics is available, it is important to check the data’s quality. Otherwise, it is counterproductive to validate a model against a unreliable dataset. For this reason, an experiment is verified into three steps:
  a. *Check experiment.* Syntactic and semantic checks of the data aimed to identify gross errors.
  b. *Validate experiment.* An expert, using its domain experience, looks for undetectable errors with automated procedures.
  c. *Experiments data cleaning.* This task is the assessment of the consistency of the experimental data respect to the predictive model. A significant difference between the experimental data and the simulated data coming from the predictive model is a precious source of information for different aspects. In other words, we can use this consistency check for a data cleaning procedure.

Similarly, the *predictive model development process* utilizes the experiments and involves three macro steps as follows:

1. *Simulate experiment.* Once an experiment is available, it is simulated with a predictive model. In particular, we can distinguish two stages:
  a. *Preparation.* It is necessary to prepare specific input files for each experiment in a format comprehensible by the simulator, specifying all the characteristics necessary to simulate the experiment accurately.
  b. *Execution.* The experiment is simulated, and the results are collected.
2. *Validate Model.* This step involves comparing the results of the model with the experimental results. This procedure can take place in two ways:
  a. *Qualitative*, involving a qualitative comparison of the results by the expert.
  b. *Quantitative*, involving the use of quantitative tools that are able to establish the similarity between an experimental curve and the simulated data.
3. *Improve predictive model.* Once the validation results are available, we need to improve the model to cover the gap from the experimental data. The predictive model for the combustion kinetics is hierarchical and modular. This feature simplifies the simulation and allows researchers to work independently on more modules at the same time. Each module covers a portion of the domain regarding a specific fuel with precise time scales and quantities. The following steps are:
  a. *Module selection.* Various reasons can bring to the decision of which modules of the model to improve, including social or industrial contexts or the availability of new experiments on a given fuel are the main reasons.
  b. *Theoretical study.* The model’s theoretical development begins, trying to understand which reactions occur, estimating or calculating the constants of the reactions depending on the problem’s complexity.
  c. *Integration.* The developed module is translated into the simulator model format and integrated into the combustion kinetics model.

Each activity requires different levels of knowledge of the domain, and for this reason, theoretically, different roles can be identified within the process. However, since there is no exact and fixed mapping between the qualification of users (student, intern, researcher, etc.) and a role responsible for a task, fine-grain privileges, that allow accessing and using each microservice to perform a particular operation in the system are defined and then assigned every time to each user based on its duties.

### 3.4 SciExpeM Framework

SciExpeM is a scientific framework that speeds up and supports the development of scientific models (cfr. Scalia et al. (2019)). The usual process, described in Section 3.3, is costly and time-consuming because the development of a model involves, for example, managing large amounts of data, especially in the predictive model validation phase. The previous sections presented the typical scientific experiment management issues that the SciExpeM framework wants to solve. SciExpeM includes a repository of scientific experiments, but its microservices architecture also offers functionalities related to the development, simulation, and analysis of scientific models.

The main goals of SciExpeM are:

• Management of scientific experiments with attention to maintaining a high quality of the repository, checking the semantic and syntax of an experiment, and an automatic semantic interpretation of the experiment for correct use of all downstream services.
• An easily extendable service architecture with the possibility of managing the user permissions.
• Automated analysis procedures aimed to provide indications and support in the development of scientific models, improve the database quality, while optimizing the use of resources.

In Section 3.4.1, we present the general approach to the problem that can be easily applied to different domains, and in Section 3.4.2 we illustrate the architecture necessary to satisfy the system’s characteristics.

#### 3.4.1 Description of the General Method

**FIGURE 2:**
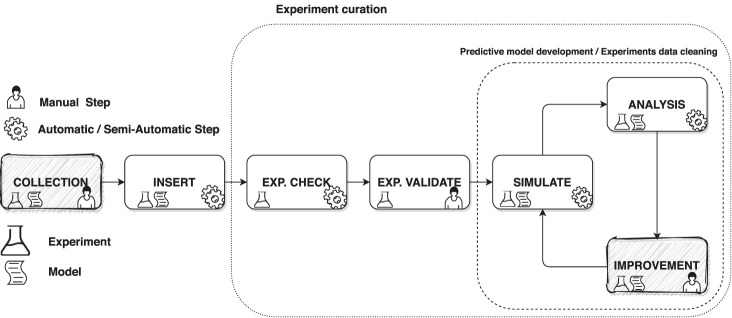
A sketch of the logical workflow of the development process of a scientific model that involves the continuous interaction with experiments. Filled in grey are the steps that are not a subject of the SciExpeM framework but are part of the overall process.

In scientific research, having a quality database is of primary importance. It is even more critical when a model development process is a data-driven approach in which experimental data play a critical role in the definition and validation of the model itself. For this reason, it is essential to have a system that can handle large amounts of scientific data in the most automated way possible. First of all, it is necessary to identify the main phases that involve a scientific experiment in the development of a predictive model. As presented in Section 3.3, the workflow of the experiments and the predictive models are closely linked and in some parts they overlap. Therefore, we summarize the overall process into seven stages. The phases are *Collection*, *Insert*, *Check*, *Validate*, *Simulate*, *Analysis*, and *Improvement* as we can see in Figure 2. Identifying the logical workflow in which an experiment is involved in the model development allows us to understand what services are required, organize the architecture, and identify the tools needed to implement the system. Furthermore, logically organizing the life cycle of experiments and the model into phases helps decoupling the operations performed on each stage’s data, defining interchangeable and extensible macroblocks.

*Collection* is a step of the overall process outside the scope of this work in which experiments and models are collected from the literature or other sources. The *Insert* phase considers all aspects of the experimental and predictive model database’s enrichment, integrating different insertion methods, even from different sources and formats. The *Check* phase limits, as far as possible, potential sources of errors in the experiments. Subsequently, in the *Validate* step, a domain expert verifies the experiment entered and provides additional information that allows the system to interpret it automatically in the subsequent phases. The *Simulation* phase represents the execution of a numerical tool with the use of a numerical model to replicate experimental data. The *Analysis* phase can be automated or customized and compares the results obtained during simulation with other information from the database. Finally, during the *Improvement* step, the model or the experimental database quality is enhanced using the results of the analysis. Also, this last phase, as for *Collection*, is outside the scope of this paper since it is not interesting for us how the predictive model is improved or which is the criterion to exclude (clean) an experiment from the process.

#### 3.4.2 Advanced Architecture

**FIGURE 3:**
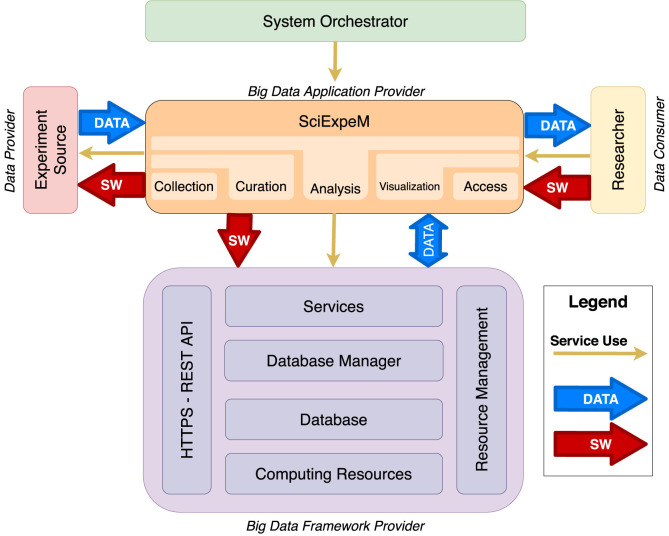
Sketch of the architecture adapted from the NIST Big Data Reference Architecture (cfr. Chang et al. (2019)) for the management of experiments to support the development of scientific models.

NIST Big Data Public Working Group in the work of Chang et al. (2019) presents the NIST Big Data Reference Architecture (NBDRA) guide that describes, using a functional component view, the roles with their actions and the components that carry out the activities for a Big Data architecture. According to these guidelines, we present in Figure 3 a general overview of the updated SciExpeM architecture.

As we can see in Figure 3, the *System Orchestrator* takes care of the configuration and management of the other components of the Big Data architecture.

The component *SciExpeM*, according to the figure and Chang et al. (2019), represents the *Big Data Application Provider* that encodes the business logic and executes a specific set of operations to the data. This entity manages the life cycle of experiments and predictive models with a different application to fulfill the requirements given by the *System Orchestrator*.

The union of four submodules represents the *Big Data Framework Provider*, with an additional transversal layer representing the Hypertext Transfer Protocol Secure (HTTPS) REST Application Program Interface (API) communication mode with the SciExpeM framework and a module of resource management and optimization. Beginning from the bottom to get to the top, we observe from Figure 3 a layered structure of the framework, in which, starting from the resources in terms of platforms such as *Computing Resources* and the *Database*, we arrive at the tools such as the *Database Manager* and the *Services* that provide the system functionalities.

Finally, the *Experiment Source* represent the system’s *Data Provider* in terms of both literature, private communications between research labs, or experiments entered by users. *Researcher* is the *Data Consumer*, representing all the user profiles that can be derived from Section 3.3, and can interface with the system to manage it or perform tasks. The system’s interaction can occur through a user-friendly interface or using the API.

## 4 Results: Realization of the SciExpeM Framework

This section illustrates the main elements of the design and the realization of the SciExpeM system, and it presents the primary services that allow the SciExpeM framework to support a predictive model’s development.

**FIGURE 4:**
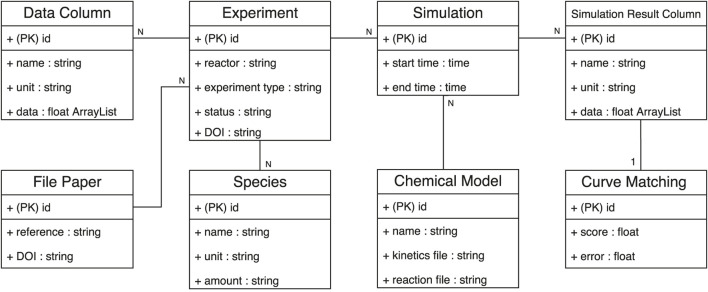
Class diagram of the database used in SciExpeM to represent experiment, simulation, models, and analyses (PK) stands for *primary key*. Some fields of the entities can be omitted for simplicity.

Before defining the services that characterize the SciExpeM framework, we briefly introduce the *operational database* structure used to represent the domain on which most services rely. Figure 4 shows the class diagram model that represents the combustion kinetics domain. This database model presents just the essential elements that allow representing sufficiently the combustion kinetics domain. We suggest evaluating the database model’s structure and complexity before implementing a system to represent a scientific domain. Having additional but not essential details burdens data management without bringing benefits in the strict sense to data-driven research. Another feature of the class diagram is that the association between the *Data Column* and *Simulation Result Column* requires additional knowledge not representable in a Database. The combustion kinetic domain is heterogeneous and complex, and representing it entirely with a defined set of rules is not easy. SciExpeM encodes this knowledge through a specific service (Section 4.1) to provide the correct mapping between the experimental data (*Data Column*) and the model simulation results (*Simulation Result Column*).

Regarding combustion kinetics, it is possible to read the class diagram in Figure 4 in the following way: an *Experiment* is an assortment of measures (*Data Column*) regarding a specific fuel mixture of *Species* collected with precise equipment and procedure. An experiment is internally identify using the *id*, but it is also mandatory a DOI of the file that represents the *Experiment* in a specific format. Moreover, an *Experiment* has a *status* field that keeps track of the *Experiment* process stage (Possible statuses are *new*, *verified*, *invalid*, etc.). Using a predictive *Chemical Model* it is possible to simulate an *Experiment* to replicate the same experimental results. For this reason, when a *Simulation* is concluded, the corresponding measures (*Data Column*) of the *Experiment* are stored in a *Simulation Result Column*, and for each pair, a similarity index is computed and saved into the *Curve Matching* for future analyses.

**FIGURE 5:**
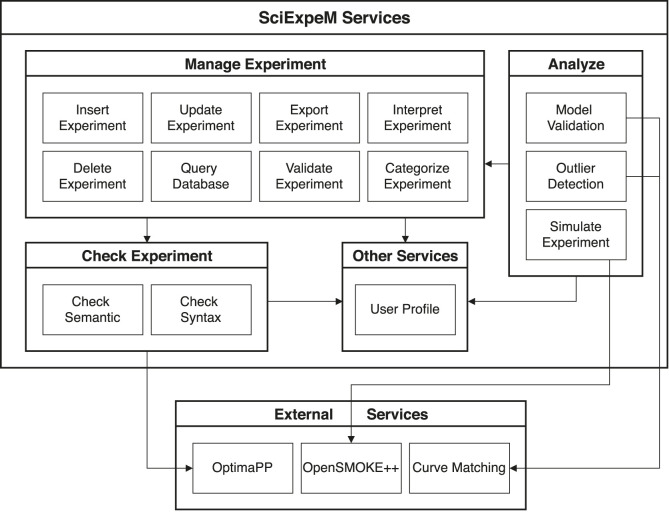
Main experiment related services of SciExpeM system with their dependencies.

Thanks to a modular service structure, as we can see in Figure 5, we can easily extend the framework capabilities by introducing additional modules and exposing new services through new endpoints in the API. The figure is a zoom-in of the services in Figure 3 and shows the dependencies among the functionalities represented by an arrow, persuing the decoupling and reuse principle of a microservices structure as explained by Alshuqayran et al. (2016) and Kratzke and Quint (2017). A benefit of SciExpeM derives directly from this approach: providing essential services to the end-users to combine them as they prefer.

Each subsection details a group of services that faces a problem with the corresponding solution. In the implementation phase, the services should be developed according to their application domain characteristics, and in some cases, they may be optional. In particular, we discuss:

1. *Manage Experiment* (Section 4.1): This group of services takes care of the management of experiments during their life cycle.
2. *Check Experiment* (Section 4.2): This collection of services tries to keep high quality in the SciExpeM scientific repository.
3. *Analyze* (Section 4.3): This group of services offers functionalities to investigate a predictive model or an experiment in different ways to improve the accuracy and the repository quality, respectively.
4. *Other Services* (Section 4.4): This block of services represents all the other services implemented by SciExpeM.
5. *External Services* (Section 4.5): This collection of services are external functionalities that are not implemented by us, but SciExpeM uses their process to complete other services.

Access to SciExpeM services needs to be as easy as possible. Providing a communication interface through endpoints is very flexible but may not be accessible for all users. For this reason, two other ways of interacting with the system have been developed that use the pre-existing endpoints. A user can access the system through a web interface that allows, for example, new experiments’ insertion. Otherwise, a user can use a Python library that maps the Database entities presented in Figure 4 into Python objects providing flexibility in implementing custom extensions.

### 4.1 Manage Experiment

This collection of services represents the system’s functionalities to manage an experiment’s life cycle.

The first step of an experiment within SciExpeM is the insertion represented by the *Insert Experiment* service. This service must take into account that the import of scientific data can take place from different formats. However, it is essential to ensure a unique representation format for the system’s experiments by choosing a widely accepted format. In the case of combustion kinetics is ReSpecTh. In this way, the system should only implement the translation into the main format without changing its subsequent interpretation from the primary format to the database schema. Besides, this approach allows for the easy export of the experiment from the system to other environments through the *Export Experiment* service. Therefore, each experiment in the system has an associated file in a commonly accepted representation format. For this reason, it is possible to associate to this file a unique DOI. Two things can happen during the insertion of a new experiment: the experiment is provided in the main representation format with an associated DOI or not. In the latter case, SciExpeM, after a user inserts an experiment through the interactive form or in a different format (for example ChemKED), generates the ReSpecTh file for the experiment, and at the right time, generates the DOI for the file. Finally, the experiment status is changed to *new* to highlight the necessity of subsequent validation by an expert.

Once an experiment is in the Database, it is important to offer a series of services for its maintenance and consultation. *Update Experiment* and *Delete Experiment* represent the services, respectively, to update or delete an experiment from the system. However, the use of this type of service is risky. Updating an experiment is intended to correct errors entered during the insertion phase and does not modify the experiment itself. Since a file with a Digital Object Identifier (DOI) is associated with an experiment, any modification would invalidate the correspondence between the experiment and the file with the DOI. On the other hand, removing an experiment from the system could affect the completeness of the predictive model validation since an experiment could be the only experimental data that cover a specific portion of the domain.

If there is a need to modify an experiment in the Database that already has an associated DOI to the corresponding ReSpecTh file, we need to apply the changes to the experiment and generate a new ReSpecTh file and associate a new DOI. For this purpose, we use Zenodo (cfr. European Organization For Nuclear Research and OpenAIRE (2013)). Zenodo generates a DOI for each file that, in our case, is a ReSpecTh file that represents an experiment. Using its DOI versioning functionalities, we can update the same record with a new version and a new DOI while keeping a separate DOI for all the older versions or a unique DOI to refer them all. In other words, we could monitor the evolution of our dataset, but in our system, we keep only the last version of the experiment. Any addition or modification in the Database regarding the experiments activates the *Check Experiment* service (Section 4.2). *Query Database* is the general service to query the system to retrieve experiments, executions, models, and curve matching scores.

Automatic checks provided by *Check Experiment* reduce the possibility of errors, but they cannot be removed entirely due to the problem’s complexity. If an experiment does not pass the checks it is rejected. Otherwise, an expert has to validate the experiment inserted with *Validate Experiment* service. This service generates the ReSpecTh file and the DOI for the experiment using Zenodo, if not present, and changes the experiment status to *verified*. Having a dataset of validated experiments is a critical aspect. It allows us to rely more on experimental data and use them to analyze and validate simulation models. During validation, the expert can add other information that helps categorizing it and having an entire comprehension of the experiment by the SciExpeM system.

Once an experiment is validated, it becomes available for a variety of further uses within the system. However, to automate this experiment’s use and analysis, it needs to be understood by the system. SciExpeM must apprehend the experiment to know which specific module of the predictive model to use and what information from the simulator it should extract to compare the simulated data with the experimental ones. This seemingly logical and straightforward problem but is not easy to automate in a complex domain. Each scientific experiment has its particularities, and it is even often difficult to associate them with a type of experiment. Another requirement to be satisfied with the implementation phase is to decouple the expertise part from the code implementation part. For this reason, SciExpeM uses a dynamic interpretation through *Interpret Experiment* service. Once the experts have defined an experiment’s discriminating characteristics, it is possible to complete a table that specifies how the system must interpret an experiment in terms of the specific solver for the simulation and interpret its results correctly. In particular, the table specifies, given the experiment’s discriminant properties, a mapping of the experimental data’s information with the simulator output information. In this way, the experts can update the table that helps the system to interpret the experiments without any changes in the platform’s source code.

Finally, *Categorize Experiment* helps during the analysis phase of the model. Scientific models try to simulate very complicated domains. For this reason, it is necessary to use elaborate scientific models to represent the problem entirely. Since the model deals with representing all possible experiments in the domain, it is interesting to understand its behavior in more specific cases. Each scientific experiment is unique but is part of a family of experiments. An experiment family can be determined by different factors depending on the application. These characteristics can be computed from the information already present in the experiment, alternatively, they can be specified by an expert. SciExpeM associates metadata to each experiment that help the system in the analysis phase. Using the categorization of the experiment, it is possible, for example, to measure the performance of a model on a specific portion of the Database, improving the comprehension of the effectiveness of a model adjustment.

### 4.2 Check Experiment

A newly inserted data may be syntactically or semantically incorrect. In this case, the system refuses the experiment and notifies the user. To verify these errors it is necessary to carry out a series of checks that guarantee a higher experiment repository quality. In an experimental context, all information or properties must be present to characterize an experiment, that defines its syntax. However, it is also necessary to carry out some basic checks on the semantics of the data entered, resulting from typing or domain-specific errors. The system performs the checks every time a service interacts with the database to add or update an experiment. The controls should happen automatically and transparently without the necessity to invoke them explicitly. SciExpeM leverages, to implement these features, the database management layer’s presence in the architecture that virtualizes the database and intercepts any interaction between the system and the database. To ensure a syntactic check over the experiment database, SciExpeM integrates OptimaPP, a software that controls whether the requirements to define a combustion kinetics experiment in ReSpecTh format is fulfilled (cfr. Varga (2020)). Regarding the semantics of an experiment, the system controls some essential elements, such as, for instance, the congruence between the unit of measurement and the declared property, or other simple experiment consistency checks.

### 4.3 Analyze Model

Providing analysis tools is fundamental to support the development process of predictive models.

**FIGURE 6:**
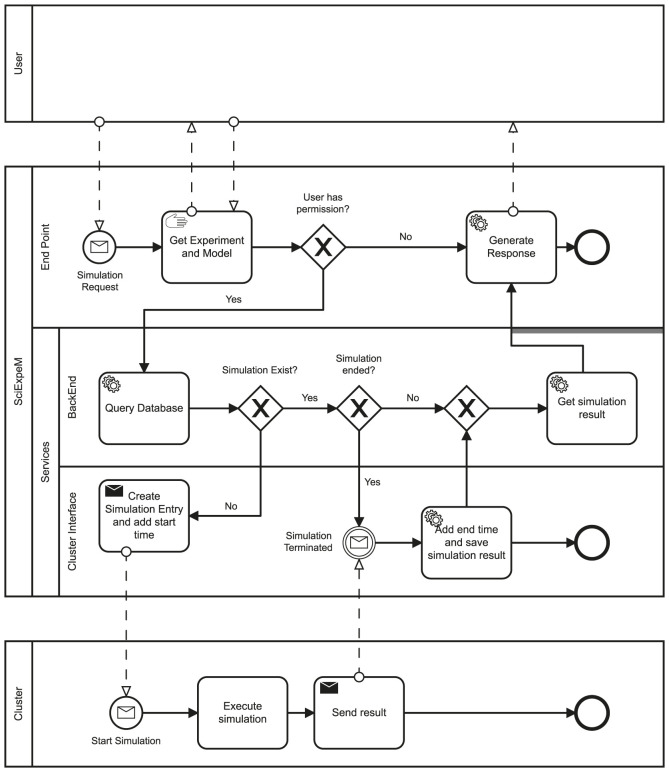
Business Process Model and Notation (BPMN) for the request of an experiment simulation. We can observe the interaction of the various SciExpeM services to fulfill the requirements about the reuse of resources and a non blocking request.

The first step in this procedure is to be able to use the *Simulate Experiment* service, described in Figure 5, to simulate an experiment through a predictive model. The services presented in Section 4.1 ensure that an experiment is *verified*, i.e., an expert has checked the experiment validity, and SciExpeM has all the information to simulate it. Simulating an experiment also requires knowing the specific model to use. The user provides this information in case of a specific request or, automatically, against all the system models. Simulating an experiment can take anywhere from a few seconds to several days. For this reason, it is crucial to fulfill two requirements. First, the simulation of an experiment must not be a blocking request. Second, it is also necessary to verify that a simulation has not already occurred or started to reuse a previous result and save resources. Figure 6 shows a Business Process Model and Notation (BPMN) that describes the interaction between various services and entities to perform a simulation fulfilling the previous requirements. When a user submits a request to start a simulation, the system creates a transaction. Within the atomic transaction, SciExpeM checks that there are no other simulations already completed or started. In any case, the system replies with the result if available, otherwise with a response containing information regarding the simulation’s status. If the request regards a new simulation, the system starts it after updating the corresponding *starting time* field of the Database’s entry. When the external process finishes the simulation’s execution, the system saves the results and updates the simulation status regarding the *ending time*. From now on, the simulation results are available.

The second step is to compare the simulation results with the experimental data to measure the scientific model’s performance using the *Validate Model* service in Figure 5. This service provides a general overview of the model performance using a quantitative tool like *Curve Matching* to measure the difference between the model’s simulation results and the experimental information as presented in Section 3.2.1. SciExpeM offers other analysis tools like *Outlier Detection*. This kind of service wants to help the improvement process of a scientific model differently. Instead of providing information regarding a model’s general performance, it gives more precise information regarding the model’s anomalous behavior in some specific cases. Since the scientific model for combustion kinetics is hierarchical and modular, *Outlier Detection* works very well with the *Categorize Experiment* service detecting precisely possible module defects. SciExpeM can automatically carry out these analyses using the *Interpret Experiment* service that provides the necessary knowledge to compare the simulator’s results with the experimental data correctly.

### 4.4 Other Services

SciExpeM relies on other services that work under the hood providing additional functionalities to the framework to support, for example, the user interface, other services, or the control over the system itself.

The *User profile* is a critical aspect of the system since it maps a research group’s roles in the permission to use a SciExpeM service. SciExpem provides authentication and service permission management to guarantee a secure working environment, reliable analysis results, and correct use of the resources. The design strategy to offer this functionality is to request specific permission for each service. In such a way, when SciExpeM offers a new service, it is necessary to associate to it a permission and then add the corresponding authorization to users that need access. A user can log in to the system through the web interface or use its authentication token and attach it to any HTTPS request to leverage the SciExpeM API.

Finally, among the *Other Services* there is a logger functionality that helps monitoring an experiment’s history and the user’s activity, in such a way it is easier to rollback a wrong operation or discover a glitch in the system.

### 4.5 External Services

SciExpeM uses a set of external services to be able to complete its services. These services are performed directly by SciExpeM but on a different process, after which the results are collected. In this case, SciExpeM uses OpenSMOKE++ (cfr. Cuoci et al. (2015)) as the simulator of the experiments, OptimaPP by Varga (2020) to check the syntax of the experiments in the ReSpecTh format and the Curve Matching to compare the curves. Using this modular service approach allows SciExpeM to manage services independently.

### 4.6 Implementation

The new implementation is based on the first version of SciExpeM (Scalia et al. (2019)). The SciExpeM system is developed using a PostgreSQL relational database to which a back end in Django is interfaced. We use PostgreSQL extensions to support complex data types not available in standard SQL, such as lists. Django exposes an HTTPS API that allows interactions with the system through a user interface in React. js. The transmission of data through the API has been optimized by compressing the server’s responses. In addition, a Python library that wraps the API and allows personalizing the analysis with Jupyter Notebooks is provided.

## 5 Preliminary Evaluation

In this preliminary evaluation, we combine the services offered by SciExpeM into use cases to illustrate the potential of the system and the benefits in the model development process with respect to a manual procedure. SciExpeM, with its services, can be an active part of the model development process, helping in the experiments management and providing analysis tools while saving time. One of its notable contributions regards model validation. Evaluating a model against a set of experiments is more exhaustive than comparing a single experiment-simulation result pair independently, enhancing the model comprehensiveness and reliability. As described in Section 3.2, a manual benchmark of the model is not suitable due to the growing number of experiments. This limitation forces the researchers to cut down the test set of experiments against which to examine the model, losing useful information with respect to a broader test set. SciExpeM overcomes these limitations and also allows a user to select a test set with specific characteristics leveraging the categorization service and reusing previous analyzes, if available. Furthermore, every time an experiment enters the SciExpeM repository, the experiment is transparently included in the scientific model’s validation-simulation-improvement loop.

In these analyses, we used the ReSpecTh repository experiments that currently contain 2,397 experiments. More precisely, we used a subset of ReSpecTh regarding the IDT of four species: Hydrogen, Carbon Monoxide, Methanol, and Ethanol.

The experiments are available online in the ReSpecTh Kinetics Data Format, and they were integrated into SciExpeM through the Insert and Check experiment services. After that, SciExpeM used the Validate and Interpret experiment services to simulate the experiments with the OpenSMOKE++ simulator described in Cuoci et al. (2015) and compared the simulated to the experimental results using the Curve Matching framework described in Section 3.2.1.

In this section, we illustrate two types of analysis in which we easily compare the model performance (Section 5.1) and automatically extract knowledge from the data (Section 5.2).

### 5.1 Model Comparison

The evolution process of a scientific model is a continuous cycle of improvement and validation, as explained in Section 3.2. It is necessary to use quantitative tools to compare the models’ results and the experimental results to validate a scientific model. Section 3.2.1 introduced Curve matching as a score of similarity, among whose characteristics it can consider the uncertainty present in the experimental data. Curve Matching is able to compare curves, trends as a whole, rather than just as a collection of points, such in case of mean square error. In practice, for each experiment we measure the similarity respect to the simulated data with a certain predictive model. For this goal, Curve matching plays a central role in our system since it is an objective tool, which requires little domain knowledge in using it, and for this reason, it lends itself very well to automated procedures.

In this case, researchers use SciExpeM to determine an improvement or worsening of a model in its different versions during its evolution. SciExpeM uses Curve Matching results to determine if there is an improvement between one model and another; in this case, the focus is not on the absolute value of the score but on its variation, negative or positive. However, it is not always immediate to conclude whether there has been a variation in the model score because the experimental data are strongly affected by uncertainty.

**FIGURE 7:**
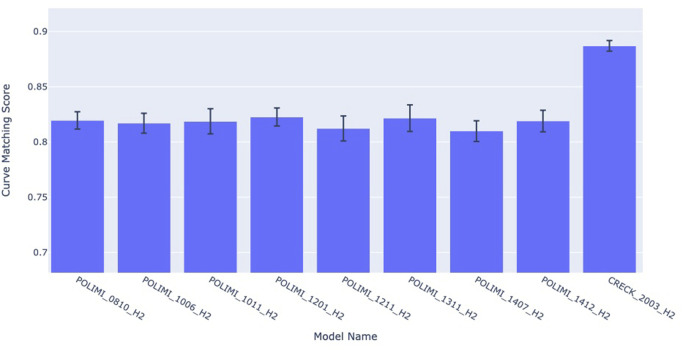
Combustion kinetics model comparison for an experiment (cfr. Chaumeix et al. (2007)) using Curve Matching as similarity score.

In Figure 7 there is an example of this analysis.

A researcher used experiment DOI:10.24388/g00000002 (cfr. Chaumeix et al. (2007)) as a benchmark. In the figure, it is possible to observe that the different versions of the model, on the *x*-axis, have different curve matching scores on the *y*-axis, but are within the uncertainty range. For this reason, most of the models have similar performances for this experiments, except for the CRECK 2003 H2 model, released in March 2020, which exhibits a significant improvement over the other models. SciExpeM, through its services, speeds up this quantitative analysis, reusing previous results and helps researchers understanding that some improvements between some different versions of the model do not bring any sensible advantage for this specific experiment, increasing the overall model comprehension.

### 5.2 Outliers Detection

In this case study, we want to illustrate how SciExpeM fits into the model refinement cycle using analysis services such as outlier detection. Firstly, using SciExpeM’s categorization service, we can select a collection of experiments with specific characteristics representing a portion of the domain and the model. In this case, four species of fuels are selected for which IDT is measured. These experiments are then automatically simulated with different models and compared with the experimental data. SciExpeM measures a series of simple statistical parameters, such as mean and variance, to determine if the model validated on this data collection exhibits anomalies. These anomalies can be identified with statistical measures assuming that the model performs similarly for experiments belonging to the same category or collection. For this reason, a drastic variation of measures suggests a problem in the model or in the repository.

In combustion kinetics experiments, an outlier could be a point in the experimental data with a significant error of measurement or a simple typo during the insertion of the data.

**FIGURE 8:**
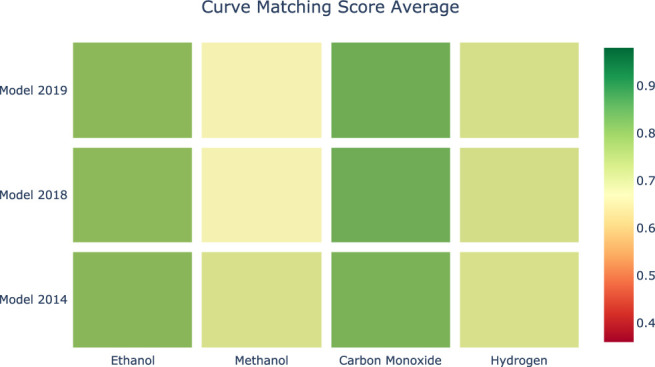
Heatmap of the average Curve Matching scores for each combination of species-model. Higher is better.

**FIGURE 9:**
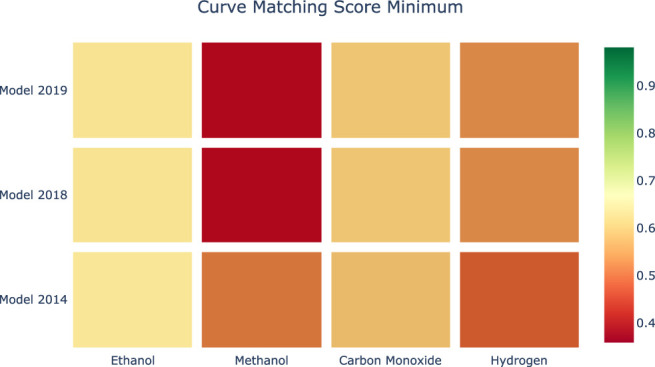
Heatmap of the minimum Curve Matching scores for each combination of species-model. Higher is better.

Figures 8, 9 provide a visual representation of the results of applying Curve Matching over a selected set of experiments for four fuels using three different models. In particular, the heatmap in Figure 8 shows the average Curve Matching scores for each model and each fuel, while Figure 9 presents the minimum Curve Matching scores. Both figures highlight the possible presence of outliers in the case of methanol.

**FIGURE 10:**
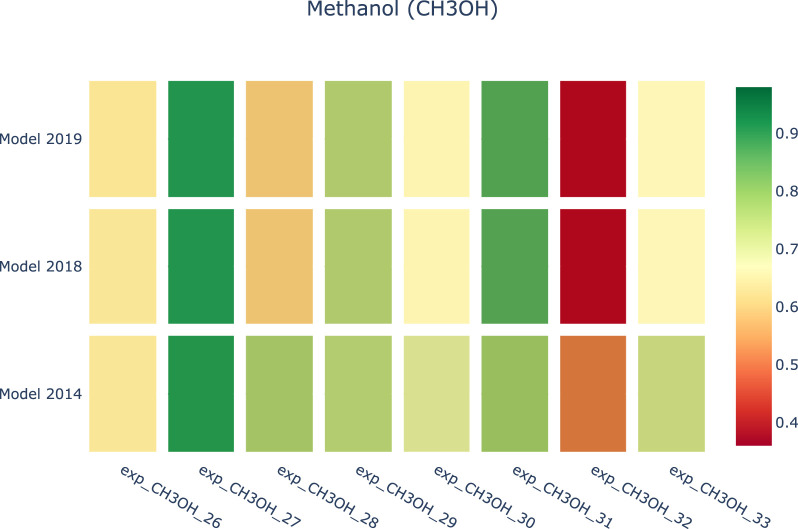
Heatmap of the Curve Matching scores against three combustion kinetic models regarding a collection of Methanol experiments.

A researcher can deeply investigate the origin of this difference. In particular, we can observe a worsening of the model performance in newer kinetic models for the methanol experiments. Figure 10 shows a drill down into the details of the methanol analyses to support the investigation of the problem source. As we can see, the major effects are due to a specific experiment. At this point, the researcher evaluates the specific case.

Usually, two cases can occur: the first consists of isolating the experiment as it contains inaccurate or unreliable information. This may be due to a variety of reasons, which can systematically corrupt the experimental dataset, e.g., i) an incorrect tuning of the instrumentation, ii) a malfunction of one of the component of the facilities, or an iii) error in the transcription of the numerical values. The second involves improving the kinetic model. Afterwards, the development process can restart its cycle. In the case of Figure 10, it is possible to observe that the update of the kinetic model from 2014 to 2019 has caused a significant worsening of the score of *exp_CH3OH_32*, which now is flagged as red. At this point, SciExpeM allows investigating more deeply on the single fuel, and human intervention can understand the source of the low index. First of all, the different contributions to the Curve Matching index (distance, similarity, and shift indices—cfr. Section 3.2.1) are isolated, and compared with the previous values. Then, experiments performed in comparable operating conditions are sought in the database to check whether the model systematically fails in such a region. At this point, if the bad performance of the model is not systematic, the single experiment can be classified as unreliable, and/or further experiments might need to be found in the literature (if available) in such region. On the contrary, if the model systematically fails in such a region, SciExpeM can identify an issue in the kinetic model and highlight the need for further theoretical research in such an operating range.

## 6 Discussion

SciExpeM proposes the use of a shared repository of experiments and of simulation results with different models. *Shared repositories* have been proposed in the literature by several groups and are being discussed at the strategical level (e.g., by NIST and in the SmartCAT COST Action^6^ for combustion) as they present several advantages to the scientific community to enable sharing and reusing of experimental data in a systematic way. As far as data storage is concerned, we observe the advantages of a database model that is simple and functional to research activities, without overly complicating the representation of the domain and with automatic checks on data quality. It is also advisable to store the data according to a widely recognized experiment format, thus facilitating data processing and analysis, and the integration of third-party libraries.

On the other hand, several open challenges mark the long-term development of such repositories. First of all, as discussed by Curry and Sheth (2018), it is necessary to clearly define the type and structure of the *community* involved. Repositories can involve many communities and potentially become virtual environments. In the present phase, SciExpeM focuses on providing a common structure to share experiments and an analysis basis for the evolution of a single model, as shown for instance in Section 5. Other repositories support multiple different types of models, usually on specific problems, such as in CloudFlame. It is necessary for a wider scope of repositories to provide a clear view on the *semantics of data* contained in the repository. Currently, in the field, there is no common shared ontology for the main concepts, and several perspectives should have to be harmonized and used in parallel, including the different formats used to represent reactions, such as molecular formulas, canonical SMILES as described in Weininger (1988), InChI as defined by Heller et al. (2015), 2-D structures and 3-D conformers.

One of the specific features of SciExpeM is the possibility of *reusing results* of simulations. In addition to performing and using a simulation for a specific research goal, the simulation results will enable a more general analysis of stored experiments and models, comparing them in a broader perspective and avoiding the repetition of experiments and simulations when they are already available. For such results to be useful, the associated metadata must be defined clearly and without ambiguities, thus effectively defining the experiment’s context. Ambiguities may arise in default values (e.g., atmospheric pressure) or default structures (e.g., not considering tautomerisms, i.e., different spatial configurations of the considered species in a reaction).

A further issue is related to *experiment management*. As illustrated in the process, activities such as corrections, data augmentation, and the like can be performed to improve the quality of the experiment. A clear track of such activities needs to be maintained as different interpretations might be possible and invalidate the results in some cases if not properly considered.

Although most of the procedures are automated, some steps of the procedure require manual supervision. In current Data Science life cycles, a *human-in-the-loop* approach is envisioned, in particular when deep learning techniques are used for the prediction of properties (see, e.g. Stodden (2020)). In particular, during the validation phase of the entered data (Section 3.4.1), the expert must provide additional information on the experiment to interpret, categorize or simulate it. In the future, we also want to systematically support this phase with further services but still maintain human validation and analysis where necessary.

Another aspect that has to be considered is the *development of new processes* within the general Data Science life cycle. Starting from the application domain, the different processes involved must be identified. In particular, it is necessary to identify the parts of the processes that can be easily automated. If the process requires a comparative analysis, it is important to identify qualitative analysis tools. Being a data-driven (or experiment-driven) application, starting from the processes identified above, it is necessary to identify the logical workflow and how it interacts with the other processes.

For each block of the workflow, there is a need to *identify new support services*, collecting their functional requirements, and developing the tools needed to implement them following the microservices perspective proposed for SciExpeM. To achieve this, the overall system’s architecture must be designed to be easily extendable and support access to the system in various ways. Furthermore, the system must offer functionalities for reusing resources and providing services for specific user profiles.

A framework like SciExpeM that integrates experiments and model analysis could significantly improve the development of predictive models. Using the human-in-the-loop pipeline, we continuously improve the model and the repository where each of these two entities is used to validate each other. In addition, the methodological procedure and its automation allow drastically reducing and standardizing the time needed to analyze and simulate hundreds of experiments with the new predictive model. This autonomous procedure also benefits from reducing human errors and working with a more significant number of data simultaneously, extracting systematic properties from the experimental and simulated data.

One of the next steps in this research is to transfer this experience to another experimental domain. It is not easy to estimate a priori the effort needed for this procedure, but, for example, the pipeline that we have proposed is general enough to be applied to most of the experimental domain, ensuring data quality and predictive model improvement. Regarding the actual implementation of the database and the services instead, each case requires a specific evaluation.

## 7 Concluding Remarks

The development of scientific models has always been of great interest because they can be used to represent a domain. In the case of combustion kinetics, it allows simulating the efficiency of reactors and fuels in terms of energy and pollutants without actually carrying out experiments that would otherwise be expensive in cost and time. Scientific models continuously improved by experimental data can overcome this problem. However, the experimental data are complicated to manage, not only in the combustion kinetic field. The experimental data come from decades of studies, which have used different representations with varying degrees of uncertainty and methodology. Besides, technological advancement has meant that this representation is become more complex to cover all the new aspects of the domain. In this context, it is necessary to develop a framework capable of combining semantic interpretation of a complex domain with automatic procedures to support the research. SciExpeM provides a series of data-oriented services to support and automate the predictive model development process while managing experimental data, and in this work it has been further extended, considering the experimental data life cycle and the model development life cycle, also demonstrating its effectiveness through several experiments. Frameworks such as SciExpeM will have an increasingly central role in research as they help, with their services, to efficiently manage data and models, minimizing the use of resources and accelerating model development. SciExpeM provides a basis for developing several different analysis processes for different types of users and different types of investigations of research problems. Moreover, the experimental results highlight that it is possible to extract knowledge from integrated experimental data, for example comparing different models and efficiently finding outliers.

## Data Availability

The back end SciExpeM source code for this study can be found in the github repository “sciexpem” at the link [https://github.com/edoardoramalli/sciexpem]. The front end SciExpeM source code for this study can be found in the github repository “SciExpeM_FrontEnd” at the link [https://github.com/edoardoramalli/SciExpeM_FrontEnd]. The list of DOIs of the validated experimental data that we used in SciExpeM are available at the link [http://doi.org/10.5281/zenodo.5121901]. Please contact the authors to request access to the framework website.

## Footnotes

1
https://eosc-portal.eu/

2
https://www.nist.gov/programs-projects/research-data-framework-rdaf

3
http://www.chemked.com/

4
https://cloudflame.kaust.edu.sa/

5
http://respecth.chem.elte.hu/

6
https://www.smartcats.eu/
